# A novel screen using the Reck tumor suppressor gene promoter detects both conventional and metastasis-suppressing anticancer drugs

**DOI:** 10.18632/oncotarget.136

**Published:** 2010-08-06

**Authors:** Ryuya Murai, Yoko Yoshida, Teruyuki Muraguchi, Emi Nishimoto, Yoko Morioka, Hitoshi Kitayama, Shinae Kondoh, Yoshinori Kawazoe, Masahiro Hiraoka, Motonari Uesugi, Makoto Noda

**Affiliations:** ^1^Department of Molecular Oncology, Kyoto University Graduate School of Medicine, Yoshida-Konoe-cho, Sakyo-ku, Kyoto 606-8501, Japan; ^2^Department of Radiation Oncology and Image-Applied Therapy, Kyoto University Graduate School of Medicine, Yoshida-Konoe-cho, Sakyo-ku, Kyoto 606-8501, Japan; ^3^Global COE Program, Kyoto University Graduate School of Medicine, Yoshida-Konoe-cho, Sakyo-ku, Kyoto 606-8501, Japan; ^4^Institutes for Chemical Research, Institute for Integrated Cell-Material Sciences, and Global COE Program, Kyoto University, Uji, Kyoto 611-0011, Japan

**Keywords:** anticancer drugs, Ras, Reck, SEAP, metastasis suppression, in vivo imaging

## Abstract

The membrane-anchored matrix metalloproteinase-regulator RECK is often down-regulated in various types of cancers; the levels of residual RECK in resected tumors often correlate with better prognosis. Forced expression of RECK in cancer cells suppresses tumor angiogenesis, invasion, and metastasis in xenograft models. *RECK* is therefore a promising marker for benignancy and a potential effector in cancer therapy. We established a cell line containing two transgene systems: (1) the secreted alkaline phosphatase (SEAP) gene fused to *Reck* promoter and (2) the *HRAS^12V^* oncogene driven by the Tet-off promoter system. This cell line exhibits transformed phenotype in regular medium and flat morphology with increased SEAP activity in the presence of doxycycline, allowing the assessment of *RECK*-inducing activity of chemicals in the contexts of both transformed and untransformed cells. Our pilot experiments with 880 known bioactive compounds detected 34 compounds that activate *RECK* promoter; among these, 10 were authentic anticancer drugs. Four selected compounds up-regulated endogenous RECK protein in several human cancer cell lines. The top-ranking compound, disulfiram, strongly suppressed spontaneous lung-metastasis of human fibrosarcoma cells in nude mice. Our data demonstrate the value of this screen in discovering effective cancer therapeutics.

## INTRODUCTION

Despite the long-standing efforts, the number of effective cancer therapeutics is far from sufficient [1], and the problems of drug-resistance arise in many cases [2]. Classical anticancer drugs were screened based on their activities to induce shrinkage of tumor xenografts in experimental animals. These include several categories of cytotoxic drugs such as DNA-damaging agents (e.g., bleomycin), anti-metabolites (e.g., methotrexate), DNA-modifying agents (e.g., doxorubicin), DNA topoisomerase inhibitors (e.g., camptothecin), and microtubules inhibitors (e.g., paclitaxel). Since these agents target essential functions of the cells, their effective doses are narrow-window, and serious side-effects are unavoidable in many cases [1].

More recent approaches based on knowledge on the molecular bases of carcinogenesis led to the development of a series of agents selectively targeting oncoproteins. Herceptin (trastuzumab), Gleevec (imatinib), and Avastin (bevacizumab) are among the successful examples of such molecular-targeted therapeutics [3]. By definition, these drugs are effective on tumors bearing specific genetic alterations; tumors initially sensitive to a drug may recur if second mutations convert the target oncoprotein resistant to the drug or if some other host mechanisms develop to make the cells less dependent on that particular pathway for their survival and/or proliferation [1].

Since invasion and metastasis are among the most problematic attributes of malignant tumors [4], inhibitors of these activities have also been sought. The matrix metalloproteinase (MMP) family is interesting in this regard, since some members of this large protease family (consisting of more than 20 members) play important roles in tumor angiogenesis, invasion, and metastasis [5]. Although initial clinical trials with some broad-spectrum MMP inhibitors were discouraging, more selective MMP inhibitors need to be developed and evaluated under optimized conditions [6].

*RECK* was initially identified as a transformation suppressor gene inducing flat reversion in a *v-K-ras*-transformed mouse fibroblast cell line [7,8]. *RECK* encodes a glycosylphosphatidylinositol (GPI)-anchored glycoprotein of ~125 kDa, which inhibits at least four cancer-associated MMPs, i.e., MMP-2, MMP-7, MMP-9, and MT1-MMP [7,9-11]. Although RECK is expressed ubiquitously in normal human organs, it is down-regulated in cancers of many organs including those of the lung (non-small cell type), colorectum, breast, and pancreas [12], in which the prevalent mechanism is probably epigenetic silencing rather than genetic mutations [13,14]. Clinical studies also indicate that the levels of residual RECK expression in resected tumor tissues positively correlate with survival of the patients [12]. Down-regulation of RECK is also found in the cells transformed by various oncogenes, including activated *RAS* [7,15,16]. Such down-regulation is probably essential for carcinogenesis, since forced expression of RECK in cancer cells suppresses tumor angiogenesis, invasion, and metastasis as assessed by xenograft experiments in nude mice [7,9]. These findings also imply that malignant behaviors of tumor cells may be suppressed if the dormant endogenous *RECK* in cancer cells be activated.

In the present study, we set up an assay system for the discovery of small molecules that up-regulate *Reck* promoter. Our pilot study with a small chemical library demonstrated the value of this assay system in discovering new anticancer drugs including those with anti-metastasis activity.

## RESULTS

### Establishment of the screening system

**Figure 1:**
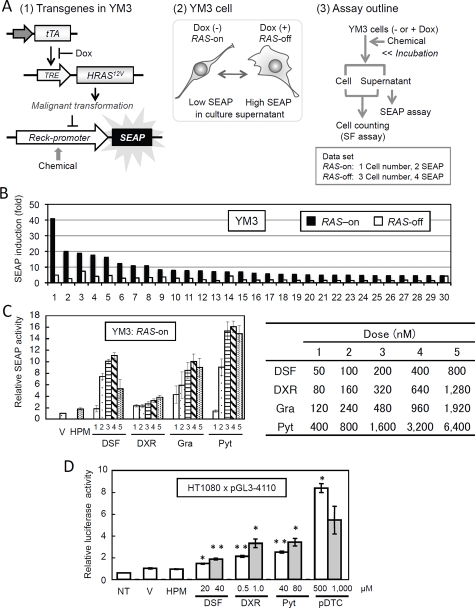
Drug screening using the Reck-promoter-reporter system. (A) Schematic representation of the transgene systems used to establish YM3 cells (1), effects of Dox on YM3 cell (2), and outline of the YM3 assay (3). (B) A part of the results of first screening. Extents of SEAP-activation (relative to untreated cells) after treatment of YM3 cells with top-34 chemicals (5 µM) for 48 h in the absence (black bars) or presence (white bars) of Dox are shown. (C) Dose-response assays using four selected chemicals on YM3 cells. Doses of each drug are listed on the right table. Data with vehicle (V; 1% DMSO) or hypothemycin (HPM; 1 µg/ml) are also shown. Bar represents mean ± SEM (n = 4). (D) Effects of four chemicals on the *Reck-luciferase* promoter in HT1080 cells. HT1080 cells stably transfected with pGL3-4110 were exposed to either DSF, Pyt, DXR, or ammonium pyrrolidine dithiocarbamate (pDTC, a compound related to DSF) at indicated doses for 48 h, and the firefly luciferase activity in the cell lysate was measured and normalized per cell. Data with vehicle (V; 1% DMSO) or HPM (1 µg/ml) are also shown. NT, no treatment. Bar represents mean ± SEM (n = 3). Student’s t-test (vs. V): * P < 0.05, ** P < 0.001.

Our previous study indicated that the 4.1-kb up-stream fragment of mouse *Reck* gene contains elements responsible for transcriptional repression by activated *RAS* oncogenes as examined by luciferase reporter assay [15]. We inserted this fragment up-stream of the secreted alkaline phosphatase (*SEAP*) gene, a marker suitable for high-throughput screening. As a recipient cell, we chose to use a derivative of rat fibroblast cell line CREF engineered to express *HRAS^12V^* oncogene under the control of tetracycline-regulated trans-activator (Tet-off) system (Fig. 1*A*, panel 1) [15], since this regulatable oncogene was expected to facilitate our initial selection of indicator cell lines that produce more SEAP when the oncogene is repressed. Moreover, such a recipient cell should allow us to compare the effects of a compound in the context of transformed and non-transformed cells (Fig. 1*A*, panel 2).

Thus, in our complete assay format (Fig. 1 *A*, panel 3), the indicator cell line named YM3 is plated onto 96-well plates in quadruplicate, two in growth medium (RAS-on) and two in the medium containing doxycycline (Dox; RAS-off), allowed to adhere for several hours, and then treated with test chemicals for 48 h. A small portion of culture supernatant was used for chemiluminescent SEAP assay, and the remaining medium and the cells were used for colorimetric quantification of viable cells. This assay yields 4 sets of duplicate data which represent the effects of chemicals on the following four parameters: (i) the number of transformed cells, (ii) the SEAP activity in transformed cells, (iii) the number of non-transformed cells, and (iv) the SEAP activity in non-transformed cells. In large-scale screenings, a simplified version using one culture condition (e.g., in the absence of Dox) can be employed, and then the selected compounds can be further evaluated using the complete format in secondary screening.

### Pilot study with a small chemical library

To test the utility of this assay in drug screening, we employed a chemical library composed of 880 structurally diverse known bioactive compounds. The biological mechanisms or pharmacological effects of these diverse compounds have experimentally been verified, and more than 85% of the compounds have been marketed either in the United States or Europe as pharmaceuticals or supplements in a wide range of therapeutic areas. In the primary screening, 151 chemicals were found to up-regulate the expression of the SEAP reporter gene more than hypothemycin (HPM), a positive control which typically up-regulates the SEAP activity about two fold by inhibiting MEK. In most cases, the extent of SEAP up-regulation (i.e., fold-induction) was higher in transformed cells than in non-transformed cells (e.g., Fig. 1*B*, black bar vs. white bar) with a few exceptions, such as chlorhexidine (Fig. 1*B*, bars 30).

We next performed dose-response studies with the 151 chemicals to validate the results of the primary screening and to determine their optimal concentrations as well as maximal activities in this assay. Thirty-four drugs that marked high scores (i.e., the extent of induction in the absence of Dox) in this secondary screening are listed in Table 1. These drugs can be classified into 8 categories based on their pharmacological activities (Table 2). Among these, four chemicals (4/34 = 12%) are tetracycline analogues (Table 1, in the “Class” column), and they probably activate the *Reck* promoter by inhibiting *HRAS12V* transactivation, which is supported by the lack of activity in the presence of Dox (Table 1, the “Activity in Dox” column). These tetracycline analogues may be considered as a set of internal controls, and their 13-fold enrichment (the original library contains 8 tetracycline analogues: 8/880 = 0.9%) unintendedly demonstrate the effectiveness of this assay.

Interestingly, eight of the 34 chemicals (8/34 = 24%; Table 1, asterisk in the “Class” column) are those included in the 88-member FDA-Approved Oncology Drugs Set (Developmental Therapeutics Program, NCI, USA), representing remarkable (12-fold) enrichment of anticancer drugs (the original library contains 18 drugs included in the FDA-Approved Oncology Drugs Set: 18/880 = 2%). Two other chemicals, camptothecine and diaziquone (Table 1, Class II with no asterisk), may also be classified as anticancer drugs. Hence, nearly one third (10/34 = 29%) of the drugs selected in this experiment represent classical anticancer drugs.

### Effects of selected chemicals on RECK expression

As materials for further studies, we selected topranking chemicals from four different pharmacological classes: disulfiram (DSF; alcohol deterrent), doxorubicin (DXR; anticancer drug), gramicidin (Gra; antibacterial agent), and pyrithione sodium salt (Pyt; antifungal agent). We first obtained fresh batches of these chemicals and confirmed their activities in two assay systems: doseresponse assay in YM3 cells (Fig. 1*C*) and luciferase reporter assay in human fibrosarcoma cell line, HT1080 (Fig. 1*D*). These results exclude the possibility that the results obtained in our pilot screening represented some artifacts due to the particular batches of compounds or to the cell line used in the screening.

**Figure 2:**
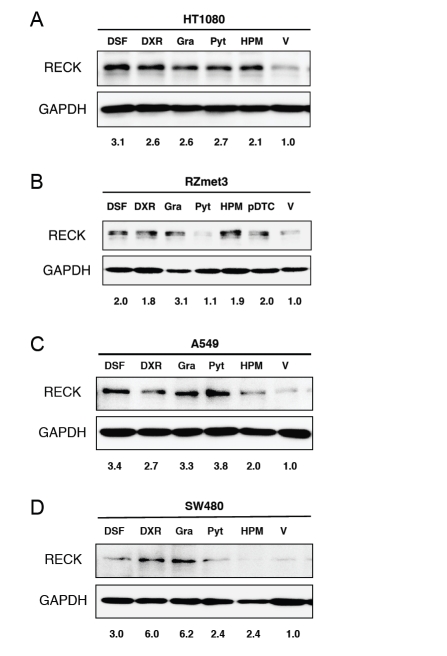
Effects of selected drugs on RECK protein expression in human tumor cell lines. The cells (density: HT1080 and A549, 5 × 10^4^; RZmet3, 1 × 10^5^; SW480, 1.5 × 10^5^ per 60-mm dish) plated on the previous day were exposed to a test chemical at IC50 as determined by colony formation assay, HPM (1 µg/ml), or 1% DMSO (V) for 48 h (A, B) or 72 h (C, D), and the cell lysates were analyzed by immunoblot assay using anti-RECK antibody (5B11D12; top panel) followed by striping and reprobing with anti-GAPDH antibody (bottom panel). Dosage: A and B (IC50 determined on HT1080), DSF 78 nM, DXR 1.8 nM, Gra 3.2 nM, Pyt 250 nM, pDTC 10 µM; C, DSF 42 nM, DXR 2.5 nM, Gra 8.4 nM, Pyt 178 nM; D, DSF 40 nM, DXR: 3.2 nM, Gra: 6.5 nM, Pyt: 170 nM. Relative band intensities, normalized against GAPDH and then divided by the normalized value for the cells treated with vehicle, are given at the bottom of each panel.

Moreover, these chemicals could up-regulate endogenous RECK protein in multiple human tumor cell lines as examined by immunoblot assay (Fig. 2), with an exception of Pyt, which showed little effects on RECK expression in RZmet3 cells under these conditions.

### Effects of DSF on tumor cells *in vitro*

Among the chemicals that marked high scores in this assay, DSF is of particular interest, since it consistently up-regulates RECK in various tumor cell lines, down-regulates gelatinases (see below), and is known to have relatively low toxicity *in vivo* [17]. We therefore chose to mainly use this drug in our subsequent studies aiming to find other bioassays useful in narrowing down promising candidates.

**Table 1.** Top-34 chemicals selected using the YM3 assay

Rank	Chemical^1)^	Optimal conc.(μM)^2)^	Induction (fold)	Activity in Dox^3)^	Class^4)^
1	**Disulfiram [DSF]**	1.67	18.0	**+**	**I**
2	**Pyrithione sodium salt [Pyt]**	1.67	17.5	**+**	**V**
3	Thimerosal	5.00	9.33	+	V
4	**Doxorubicin hydrochloride [DXR]**	5.00	8.55	+	II*
5	Camptothecine (S,+)	5.00	7.71	+	II
6	**Gramicidin [Gra]**	5.00	7.30	+	IV
7	Daunorubicin hydrochloride	1.67	6.22	+	II*
8	Cephaeline dihydrochloride heptahydrate	5.00	5.61	+	VII
9	Mechlorethamine hydrochloride	5.00	4.98	±	II*
10	Emetine dihydrochloride	1.67	3.79	-	VII
11	Mitoxantrone dihydrochloride	0.556	3.60	+	II*
12	Diaziquone	5.00	3.54	±	II
13	Haloprogin	5.00	3.48	+	V
14	Lycorine hydrochloride	5.00	3.44	+	VII
15	Methotrexate	5.00	3.42	±	II*
16	Paclitaxel	5.00	3.32	±	II*
17	Menadione	5.00	3.21	+	VIII
18	Albendazole	1.67	2.89	±	VI
19	Meclocycline sulfosalicylate	0.0617	2.59	-	IV^¶^
20	Demeclocycline hydrochloride	1.67	2.57	-	IV^¶^
21	Minocycline hydrochloride	0.556	2.54	-	IV^¶^
22	Podophyllotoxin	5.00	2.48	±	VIII
23	Harmine hydrochloride	5.00	2.44	±	VIII
24	Pyrimethamine	5.00	2.33	±	III
25	Trimeprazine tartrate	5.00	2.30	-	VIII
26	Cycloheximide	5.00	2.29	±	IV
27	Perhexiline maleate	5.00	2.28	+	VIII
28	Triamterene	5.00	2.25	-	VIII
29	Triflupromazine hydrochloride	0.0617	2.18	-	VIII
30	Raloxifene hydrochloride	0.556	2.17	-	II*
31	Piperlongumine	5.00	2.06	-	VIII
32	Hycanthone	5.00	2.02	+	VI
33	Etoposide	5.00	1.99	+	II*
34	Doxycycline hydrochloride	0.185	1.96	-	IV^¶^

1) Chemicals selected for detailed studies are in bold letters, with their abbreviations in the parentheses.

2) Optimal concentration determined by dose-response assays (dosages: 0.0617, 0.185, 0.556, 1.67, 5.00 µM) using YM3 cells.

3) Activity in the presence of Dox (i.e., HRAS^12V^-off): +, more than 1.9-fold; +, between 1.9 and 1.5-fold; −, less than 1.5-fold

4) See Table 2.

* Included in FDA-Approved Oncology Drugs Set, Developmental Therapeutics Program, NCI

¶ Tetracycline analogue

**Table 2.** Properties of the top-34 chemicals

Class	Chemical	Target of inhibition (application)/*mode of action*
I. Alcohol deterrent	**Disulfiram [DSF]**	Aldehydodehydrogenase in hepatocyte, superoxide dismutase-1, *chelation of zinc and copper cations*
II. Anticancer drugs		
Vinca alkaloid	Camptothecine (S, +)	Topo I
Podophyllum alkaloid	Etoposide	Topo II
Anthracyclines	**Doxorubicin [DXR]**	DNA synthesis, Topo II
	Daunorubicin	DNA synthesis, Topo II
Nitrogen mustard	Mechlorethamine	DNA synthesis, Topo II
DNA alkylator	Diaziquone	DNA synthesis
Antibiotic	Mitoxantrone	DNA synthesis
Taxane	Paclitaxel	Tubulin dissociation
Hormone analogue	Raloxifene	Estrogen receptor
Antimetabolite	Methotrexate	Dihydrofolate reductase
III. Antimalarial agent	Pyrimethamine	Dihydrofolate reductase
IV. Antibacterial agents		
Antibiotic peptide	**Gramicidin [Gra]**	Cell membrane, phospholipid
Piperidone	Cycloheximide	Protein synthesis, ribosome
Tetracycline analogues	Demeclocycline	Protein synthesis, bacterial ribosome
	Doxycycline	Protein synthesis, bacterial ribosome
	Meclocycline	Protein synthesis, bacterial ribosome
	Minocycline	Protein synthesis, bacterial ribosome
V. Antifungal agents	**Pyrithione sodium salt [Pyt]**	*Chelation of zinc cation*
	Haloprogin	*Damaging fungal membrane*
	Thimerosal	*Generation of ethyl-mercury*
VI. Anthelmintic agents	Albendazole	Tubulin polymerization (antiworm)
	Hycanthone	DNA/RNA synthesis, parasite nervous system (antischistosome)
VII. Vomiting alkaloids	Cephaeline	Protein synthesis
	Emetine	Protein synthesis
	Lycorine	Protein synthesis
VIII. Others	Menadione (Vitamin K3)	*ROS generation*
	Podophyllotoxin	Tubulin polymerization (antiwart, precursor of etoposide)
	Trimeprazine (Alimemazine)	Histamine receptor, muscarinic acetylcholine receptor
	Triflupromazine	Dopamine receptor, muscarinic acetylcholine receptor
	Perhexiline	L-type calcium channel, sodium/potassium ATPase, carnitine palmitoyltransferase-1
	Triamterene	Sodium/potassium ATPase
	Harmine	Monoamine oxidase A
	Piperlongumine	Thromboxane A2 receptor

**Figure 3:**
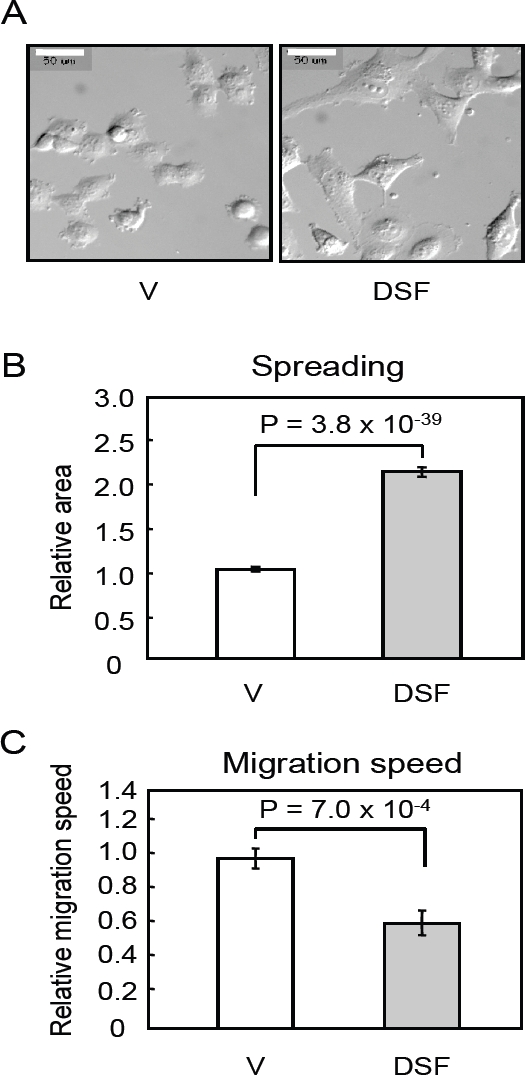
Effects of DsF on the morphology and migration of rZmet3 cells *in vitro* (A) Differential interference contrast (DIC) images of RZmet3 cells incubated in the absence (V, 1% DMSO) or presence of DSF (10 µM) for 24 h. Scale bar: 50 µm. (B) Cell spreading as assessed by the area occupied by individual cells on micrographs as shown in (A) using Image J software. The results are presented as the ratio to the control cells. Bar represents mean ± SEM (n = 100). (C) Relative speed of random migration in the absence (V, 1% DMSO) or presence of 10 µM DSF. Bar represents mean + SEM (n = 10). suppress malignancy via activation of *RECK* while some others may suppress malignancy by other mechanisms, consequently up-regulating *RECK*. This assay therefore has a feature of both molecular-targeting approach and bioactivity-oriented approach. We also expected that this assay may not detect cytotoxic drugs, since it relies of a positive readout (i.e., promoter activation in viable cells) rather than a negative one (e.g., cell killing or tumor shrinkage).

Previous studies indicate that transfection of *RECK*-expression vector induces flat reversion (i.e., increased cell-substrate adhesion) in transformed cells [7] and suppresses cell migration in several cell types [18,19]. We therefore examined the morphology and behavior of RZmet3 cells in the absence or presence of DSF by time-lapse video-microscopy (Fig. 3). DSF was found to induce flattening/spreading of these cells (Fig. 3*A, B*) and to reduce the speed in random migration (Fig. 3*C*). Hence, DSF could recapitulate some effects of *RECK*-transgene on the morphology and behavior of these sarcoma cells.

### Effects of DSF on tumor growth and metastasis

**Figure 4:**
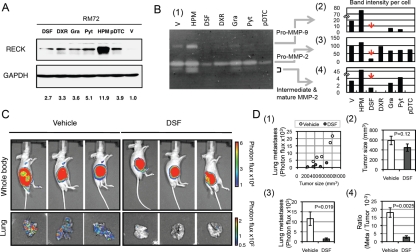
Effects of DsF on rM72 cells. (A) Effect of various chemicals on the level of endogenous RECK protein in RM72 cells. The cells (1 × 105 cells per 60-mm dish) plated on the previous day were treated with indicated drug (dosage: see legend to Fig. 2A, B) for 48 h, and the level of RECK protein was estimated by immunoblot assay. Relative band intensities, normalized against GAPDH and then divided by the value for vehicle (V; 1% DMSO), are given at the bottom. (B) Gelatin zymography with culture supernatant of RM72 cells treated with indicated chemicals for 48 h (1). Intensity of the bands corresponding to pro-MMP-9, pro-MMP-2, and intermediate/mature MMP-2 were estimated using MultiGauge software (FUJIFILM), normalized against the cell number as determined by SF assay, and presented in the separate bar graphs (2-4). Dosage: DSF (16 µM), DXR (210 nM), Gra (32 nM), Pyt (5 µM), HPM (1 µg/ml), or pDTC (20 µM). (C, D) Effects of DSF on the growth and lung metastasis of RM72 cells inoculated subcutaneously into nude mice. Nude mice bearing an RM72 tumor (diameter ~3 mm) were treated (intraperitoneal injection) with vehicle (V) or DSF (50 mg/kg/day) for 14 days. Bioluminescence flux from whole body (top panels) and the lungs (bottom panels) were recorded. Three animals from each group bearing a primary tumor of similar size are shown in (C). (D) Statistical data of nude mouse assays as shown in (C): (1) relationship between tumor volume and lung metastasis (relative bioluminescence intensity; each dot represents one animal), (2) tumor volume, (3) metastasis, (4) metastasis/tumor volume. Bar represents mean ± SEM (n = 5). Experiments were repeated twice with similar results.

Expression of RECK in HT1080 cells results in down-regulation of pro-MMP-9 and active MMP-2 in culture supernatant [7,9,20] and in suppression of metastatic activity in nude mice [7]. To facilitate the evaluation of anti-tumorigenic/anti-metastatic activity of *Reck*-activating drugs *in vivo*, we established a luciferase-tagged derivative of HT1080 (named RM72) that shows spontaneous lung-metastasis within two weeks after subcutaneous inoculation into nude mice. RM72 cells were found to respond to the four chemicals by increased RECK expression (Fig. 4*A*) and concomitant decreases in the level of pro-MMP-9 in culture supernatant (Fig. 4*B*, panel 1 and 2). In addition, DSF could also lower the levels of pro-MMP-2 as well as intermediate and mature MMP-2 in culture supernatant (Fig. 4*B*, panels 3 and 4).

Given these promising results *in vitro*, we then tested the effects of DSF on the growth and metastasis of RM72 cells in nude mice. In this assay, RM72 cells were inoculated subcutaneously into nude mice, allowed to grow into small tumor for 5 days, and then treated with DSF dissolved in olive oil (50 mg/kg/day) or vehicle alone (control) by intraperitoneal injection for 14 days (Fig. 4*C*). Under these conditions, DSF had no significant effects on the volume of primary tumors (Fig. 4*D*, panels 1 and 2), and yet lung-metastasis was strongly suppressed in the DSF-treated mice (Fig. 4*D*, panels 1 and 3). The difference is even more significant when the extent of metastasis is normalized against tumor volume (Fig. 4*D*, panels 4). No obvious side effect was observed during these experiments.

Taken together, these data demonstrate the utility of our *Reck*-promoter-reporter assay using YM3 cells for discovering promising candidates for cancer therapeutics, which include anti-metastatic drugs with low toxicity as well as conventional classes of anticancer drugs. The RM72 cells also provide a powerful assay system that allows us to rapidly assessing the effects of various agents on tumor growth and metastasis *in vivo*.

## DISCUSSION

RECK is down-regulated in a wide variety of tumors and in the cells transformation by various oncogenes [7,12,15]; forced expression of RECK in tumor cells results in suppression of tumor proliferation, angiogenesis, invasion, and metastasis [7,9]. RECK is therefore a useful marker for benignancy and a promising target (or effector) to be activated in cancer therapy. The *Reck*-promoter-reporter assay may detect compounds that suppress malignancy via multiple mechanisms: e.g., some may suppress malignancy via activation of *RECK* while some others may suppress malignancy by other mechanisms, consequently up-regulating *RECK*. This assay therefore has a feature of both molecular-targeting approach and bioactivity-oriented approach. We also expected that this assay may not detect cytotoxic drugs, since it relies of a positive readout (i.e., promoter activation in viable cells) rather than a negative one (e.g., cell killing or tumor shrinkage).

To our surprise, however, our pilot screening detected “cytotoxic” anticancer drugs very efficiently. These drugs activate *Reck* promoter while killing the cells; we also found many drugs in the library that kills the cells without activating *Reck* promoter. Hence, our finding may imply that *RECK* is a common effector for these anticancer drugs. Alternatively, these drugs may induce a common change in the cells that leads to *RECK* up-regulation. Nevertheless, this assay must be useful in finding new compounds with anticancer activities of conventional types.

Could this screen be useful in finding new types of anticancer drugs? In this particular case, could we expect anticancer activities in any of the 20 non-Class-II compounds listed in Table 1 (excluding tetracycline analogues)? One may argue that the activity to up-regulate *Reck* may be just one of several properties found in anticancer drugs so that only a fraction of *Reck*-activating drugs (e.g., Class II) represent *bona fide* anticancer drugs. Our observations with DSF, however, argue against this possibility.

DSF has been used as an anti-alcoholism drug, since it inhibits aldehydodehydrogenase in hepatocytes, leading to an acute sensitivity to alcohol [21,22]. In recent years, several papers have suggested anticancer activities of DSF (i.e., suppression of tumor growth, invasion *in vitro*, and angiogenesis and metastasis *in vivo*) [17,23-28]. Low toxicity *in vivo* distinguishes DSF from classical cytotoxic anticancer drugs [17]. Proposed mechanisms of its actions include redox-related apoptosis [23], up-regulation of metallothionein [28], inhibition of proteasome [26] or superoxide dismutase-1 activity [24], and down-regulation of several genes including *MMP2, MMP9* [25,27], *CYP2E1* [29], and *MCM* [28]. This study not only expands our knowledge on DSF by demonstrating its activities to suppress spontaneous fibrosarcoma metastasis and to up-regulate *RECK* but also strengthen our notion that the *Reck*-promoter-reporter assay may be useful in finding new types of anticancer therapeutics, including agents with low toxicity *in vivo* and with anti-metastatic activity. Some other drugs selected by this assay, such as perhexiline, triamterene (Na^+^/K^+^-ATPase inhibitors), and piperlongumine (thromboxane A2 receptor antagonist), also support this prospect, since anticancer activities of cardiac glycosides (Na^+^/K^+^-ATPase inhibitors) [30] and the involvement of thromboxane A2 signaling in angiogenesis and tumor metastasis [31,32] have been reported.

The observed effects of DSF on the behaviors of HT1080 cells in culture (Figs. 2 and 3) are consistent with the model that RECK plays an important role in DSF-mediated metastasis suppression. Our preliminary data indicate that RECK-depletion with siRNA suppresses DSF-mediated pro-MMP-9 down-regulation (data not shown), and this is consistent with the previous observation by Takagi *et al*. that RECK suppresses *MMP9* transcription [20]. It remains to be clarified, however, to what extent the up-regulated RECK contributes to the DSF-mediated metastasis suppression *in vivo*. Conditional *Reck* mutant mice may be useful in addressing this issue. In such studies, we may also be able to test the interesting possibility that DSF and *Reck* suppress tumor metastasis by regulating stem cell niche [33-35].

Given a promising assay system that can be adapted to high throughput screenings, it is also important to have independent and efficient assay systems for narrowing down promising candidates. In this study, we tested five assays, mainly using HT1080 fibrosarcoma cell line and its derivatives: (i) luciferase reporter assay (to assess the effects on the *Reck* promoter in human cells), (ii) immunoblot assay with several human tumor cell lines (to see whether endogenous RECK protein can be up-regulated), (iii) gelatin zymography with culture supernatant (to assess the effects on gelatinases), (iv) time-lapse microscopy (to assess the effects on cell morphology and motility), and (v) subcutaneous inoculation into nude mice (to assess the effects on tumor growth and metastasis). Among these assays, ii), iii) and v) seem to be useful for our purpose. YM3 assay performed in the presence of Dox is also useful in classifying the candidates into two groups based on their dependence on RAS pathway. Combination of two or more agents tested in one or more of these assays may also be useful for classifying drugs as well as for finding synergistic combinations of drugs.

Although it was an obvious option, we did not choose to use Matrigel invasion assay for narrowing down candidates, mainly because of its relatively high cost. Instead, we concentrated on establishing a reliable assay to monitor tumor growth and metastasis *in vivo*. The luciferase-tagged, highly metastatic cell line RM72 is particularly useful for this purpose, since it allows us to detect and quantify metastases as well as tumor growth in living animals within a relatively short period of time, typically in 3 weeks. Given the prevalence of carcinomas among human malignancies, assays using epithelial or carcinoma-derived cell lines may be considered more appropriate. We believe, however, that the use of mesenchymal cell lines, CREF and HT1080, as useful model systems in early steps of drug screening can be justified in light of the widely recognized importance of epithelial-mesenchymal transition in carcinoma progression [36]. Of course, the efficacy of candidate drugs should also be tested in carcinoma models in later steps of evaluation.

The chemicals detected by this assay may give us some hints on the mechanisms of *Reck* gene regulation. For instance, the list (Tables 1, 2) contains multiple compounds sharing common activities or properties, such as heavy-metal chelators, DNA-replication inhibitors, microtubules inhibitors, dihydrofolate reductase inhibitors, and protein synthesis inhibitors. In fact, many of these drugs as well as menadione [37] are potential generators of reactive oxygen species (ROS) [38-42]. The four top-ranking drugs, however, could up-regulate RECK in SW480 which carries a mutant *p53* gene [43] (Fig. 2D), suggesting p53-independent nature of this response. Further studies to understand how these drugs activate *Reck* transcription may provide fresh insights into the mechanisms of transcriptional regulation, signal transduction, malignant transformation, and its suppression.

## MATERIALS AND METHODS

### Animal experiments

The experiments using mice had been approved by Animal Research Committee, Kyoto University, and were performed in accordance with MEXT Notice No. 71 and the Act on Welfare and Management of Animals, Japan.

### Cell lines and cell culture

The origins of cell lines used are as follows: CREF, rat embryo fibroblast [44]; HT1080, human fibrosarcoma [45]; A549 human lung adenocarcinoma [46]; SW480, human colon adenocarcinoma [47]. These cells were cultured in growth medium [DMEM (Nacalai), 10% fetal bovine serum (FBS), penicillin-streptomycin] under standard conditions. RZmet3, a derivative of HT1080 carrying the *neo* marker and recovered from a lymph node metastasis after subcutaneous inoculation into a nude mouse [7], was cultured in growth medium containing 1 mg/ml G418.

### Plasmids and transfection

A segment of mouse *Reck* gene promoter [4.1 kb KpnI-HindIII fragment from pGL3-4110 [15] was inserted between the corresponding sites of pSEAP2-Basic (Clontech) to generate pSEAP-RP4.1. Stable transfection was performed using CalPhos Mammalian Transfection Kit (Clontech) with purified cellular DNA as carrier [Ratio (carrier:plasmid; w/w) = 1:3-1:5].

### Establishments of indicator cells

A derivative of CREF cell line, named TF323-C3, carrying the *HRAS^12V^* oncogene under the control of a tetracycline-sensitive transactivator (Tet-off) system has been described elsewhere [15]. TF323-C3 contains *neo* marker and exhibits transformed morphology in growth medium and flat morphology in the presence of doxycycline (Dox; 2 μg/ml). TF323-C3 cells were cotransfected with pSEAP-RP4.1 mixed with pUCSVBSD [ratio (w/w) = 4:1] followed by selection in growth medium containing 8 μg/ml blasticidine-S. A clone (named YM3) that showed highest degree of SEAP up-regulation (see below for SEAP assay) after Dox-treatment was selected. SEAP was also up-regulated in YM3 cells after treatment with hypothemycin (1 μg/ml), a MEK inhibitor inducing flat reversion in *v-K-ras*-transformed cells [48,49]. To establish an indicator cell line for assessing antitumorigenic and anit-metastatic activities of a drug in vivo, RZmet3 cells were co-transfected with pGL4 (contaning *Photinus pyralis* luciferase gene; Promega) mixed with pcDNA3.1(-)-Hygro (Invitrogen), followed by selection in growth medium containing hygromycin-B (400 U/ml). Two clones stably expressing high levels of luciferase were isolated and inoculated subcutaneously into nude mice. After 4 weeks, metastatic foci were resected from their lungs, dissociated, and cultured in growth medium containing G418 and hygromycin-B. After re-cloning in culture, one clone retaining high luciferase activity as well as lung-metastatic activity was isolated and named RM72.

### Chemicals

The 880-member Prestwick Chemical Library (Prestwick Chemical, Illkirch, France) dissolved in nano-pure grade dimethyl sulfoxide (DMSO; Wako) was used for initial screening. Independent batches of disulfiram, doxorubicin, gramicidin, pyrithione sodium salt, and ammonium pyrrolidine dithiocarbamate were obtained from Sigma. Hypothemycin was a kind gift from Shionogi & Co. Ltd.

### SEAP assay and luciferase assay

YM3 cells seeded onto 96-well plates at 1 × 10^4^ cells in 100 µl growth medium per well were incubated for 5 h to allow the cells to settle. The drug solution (500 µM, 1 µl) was added to the medium, and the plates were incubated for additional 48 h. A portion (10 µl) of culture supernatant was sampled, incubated at 65 °C for 30 min, and then subjected to the SEAP assay using Great EscAPe^TM^ SEAP Chemiluminescence Detection Kit (Takara Bio). The remaining medium and the cells were subjected to the cell-counting assay using SF reagent (Nacalai): i.e., SF reagent (10 µl per well) was added to the medium, the plates were incubated for 3 h in a CO_2_-incubator, and the absorbance at 450 nm (A_450_) was measured. The SEAP chemiluminescence count was divided by the A_450_ value to obtain SEAP activity (per cell), which was further divided by SEAP activity of the cells treated with vehicle (1% DMSO) to obtain relative SEAP activity. The primary and the secondary (doseresponse) screenings were performed in duplicate, and the validation study (with new batches of compounds) was performed in quadruplicate. For luciferase reporter assay, HT1080 cells stably transfected with pGL3-4110 [15] or pGL3-Basic (Promega) were plated onto 96-well plate (5 × 10^4^/well in 100 μl growth medium), incubated for 24 h, and 1 μl chemical solution (100-fold concentrate) or vehicle (DMSO) was added per well. After incubation for 48 h, the cells were lysed and subjected to luciferase assay using Steady-Glo Luciferase Assay Kit (Promega).

### Immunoblot assay

The cells were lysed as described previously [9], and the protein concentration was determined using DC protein assay kit (BIO-RAD). Proteins separated by SDSPAGE (10% acrylamide) were subjected to immunoblot assay using the mouse monoclonal anti-RECK antibody (5B11D12), followed by re-probing with anti-GAPDH antibody (6C5, Ambion). For visualization, the Enhanced Chemiluminescence kit (Millipore) was used with HRP-conjugated anti-mouse IgG-F (ab′)2 monoclonal antibody (Cell Signaling) as a secondary antibody. Images were recorded and analyzed using LAS-3000 and the MultiGauge software (FUJIFILM).

### Motility assay

RZmet3 cells (2 × 10^4^) seeded onto a 35-mm glass-base dish (IWAKI) were incubated for 24 h, and the medium was replaced with growth medium containing 1% DMSO (vehicle) or 10 µM disulfiram. After incubation for 24 h, the medium was replaced to Leibovitz’s L-15 (GIBCO) containing 10% FBS and 1% DMSO or 10 µM disulfiram. Cell movement was recorded by time-lapse microscopy for 3 h (3-min interval) as described elsewhere [19]. The speed of migration was calculated from a time series of coordinates (reference point: center of the nucleus) using Dunn’s formula [50]. Statistical significance was assessed by Student’s t-test.

### Gelatin zymography

Cells (2 × 10^4^/well) seeded onto 96-well plates and incubated for 24 h were exposed to fresh growth medium with or without various drugs or 1% DMSO (vehicle). After incubation for 48 h, medium was replaced to 100 µl DMEM containing 0.1% FBS and incubated for additional 12 h. The culture supernatant was harvested, cleared by centrifugation, and analyzed by gelatin zymography as described previously [7]. Band intensity was normalized against the cell number determined by SF assay.

### Tumor imaging *in vivo*

Mice were anesthetized and injected intraperitoneally with 75 mg/kg of d-luciferin (Promega) in PBS(-). Bioluminescence images were acquired with the IVIS Imaging System (Xenogen) at 5 min after injection [51,52]. Photons emitted from living mice or from isolated organs were collected and integrated for a period of 60 seconds. Images were analyzed using the Living Image software (Xenogen).

### Human fibrosarcoma therapy model in nude mice

For spontaneous metastasis assays, RM72 cells (3 × 10^6^) suspended in 0.1 ml PBS were injected subcutaneously into the right posterior flank of Balb/c nude mice (6 week old, male, Charles River). Small tumors (~3 × 3-mm diameter) developed 5 days after injection. The mice were randomly divided into two groups (n = 5 per group) and treated with DSF (50 mg/kg/day) dissolved in olive oil or olive oil alone (vehicle) via intraperitoneal injection using 24-gauge needles. After 14-d treatment, the mice were anesthetized, and their bioluminescence was recorded as described above. After the whole-body recording, the lungs were resected, washed with PBS (-), and subjected to bioluminescence recording. The tumor size (length × width × height) was measured once a week. Statistical significance was assessed by Student’s t-test.

## Abbreviations used:

MMP: matrix metalloproteinase
RECK: reversion-inducing cysteine-rich protein with Kazal motifs
Dox: doxycycline
SEAP: secreted alkaline phosphatase
DMSO: dimethyl sulfoxide
HPM: hypothemycin
DSF: disulfiram
DXR: doxorubicin
Gra: gramicidin
Pyt: pyrithione sodium salt
pDTC: pyrrolidine dithiocarbamate
GAPDH: glyceraldehyde-3-phosphate dehydrogenase
SEM: standard error of mean
